# Study on the Effect of Sn, In, and Se Co-Doping on the Thermoelectric Properties of GeTe

**DOI:** 10.3390/ma17030551

**Published:** 2024-01-23

**Authors:** Tao Guo, Guangbing Zhang, Bohang Nan, Guiying Xu, Shuo Li, Lingling Ren

**Affiliations:** 1 School of Materials Science and Engineering, University of Science and Technology Beijing, Beijing 100083, China; gt372328@163.com (T.G.);; 2 Center for Advanced Measurement Science, National Institute of Metrology, Beijing 100029, China

**Keywords:** spark plasma sintering, multiple doping, cooperative regulation, thermoelectric performance

## Abstract

GeTe and Ge_0.99-*x*_In_0.01_Sn*_x_*Te_0.94_Se_0.06_ (*x* = 0, 0.01, 0.03, and 0.06) samples were prepared by vacuum synthesis combined with spark plasma sintering (SPS). The thermoelectric properties of GeTe were coordinated by multiple doping of Sn, In, and Se. In this work, a maximum *zT*(*zT* = *S*^2^*σT*/*κ*) of 0.9 and a power factor (*PF* = *S*^2^*σ*) of 3.87 μWmm^−1^ K^−2^ were obtained in a sample of Ge_0.99_In_0.01_Te_0.94_Se_0.06_ at 723K. The XRD results at room temperature show that all samples are rhombohedral phase structures. There is a peak (~27°) of the Ge element in GeTe and the sample (*x* = 0), but it disappears after Sn doping, indicating that Sn doping can promote the dissolution of Ge. The scattering mechanism of the doped samples was calculated by the conductivity ratio method. The results show that phonon scattering Is dominant in all samples, and alloy scattering is enhanced with the increase in the Sn doping amount. In doping can introduce resonance energy levels and increase the Seebeck coefficient, and Se doping can introduce point defects to suppress phonon transmission and reduce lattice thermal conductivity. Therefore, the thermoelectric properties of samples with *x* = 0 improved. Although Sn doping will promote the dissolution of Ge precipitation, the phase transition of the samples near 580 K deteriorates the thermoelectric properties. The thermoelectric properties of Sn-doped samples improved only at room temperature to ~580 K compared with pure GeTe. The synergistic effect of multi-element doping is a comprehensive reflection of the interaction between elements rather than the sum of all the effects of single-element doping.

## 1. Introduction

Thermoelectric materials (TEs) can realize the direct conversion of thermal and electrical energy with the merits of being environmentally friendly, not having moving parts, and so on [1,2]. TEs are mainly used in two aspects, including thermoelectric power generation and thermoelectric refrigeration [3,4,5]. TE devices are especially suitable for power supply systems under extreme conditions as they can use the temperature difference to work. Radionuclide generators based on TEs have been recognized as the best power supply for long-term deep space exploration missions [6]. The energy conversion efficiency of TEs is determined by the *zT* value which can be defined as:

*zT* = *S*^2^*σT*/*κ* (1)

*PF* = *S*^2^*σ* (2)

*κ* = *κ*_*e*_ + *κ*_l_ (3)

where *PF* = *S*^2^*σ* is the power factor contributed to by the Seebeck coefficient *S* and the electrical conductivity *σ* to evaluate the electrical performance, *κ* is the total thermal conductivity contributed to by the electrical thermal conductivity *κ_e_* and lattice thermal conductivity *κ_l_*, and *T* represents the absolute temperature [7,8,9].

GeTe-based materials have been considered as promising thermoelectric materials since the 1960s, and their performance has been greatly improved recently [10,11]. GeTe belongs to a P-type narrow-band-gap semiconductor, which is the rhombic phase (R-GeTe, R3 m) below 700 K and the cubic phase (C-GeTe, Fm3m) above 700 K [12]. During the phase transition process, the eccentric atoms in R-GeTe return to the central position in C-GeTe, resulting in an increase in crystal structure symmetry.

The performance of GeTe will reach a peak value in the range of 600–800 K and is suitable for moderate-temperature applications. However, the Ge vacancy formation energy of GeTe is very low, and the formation of one Ge vacancy will produce two hole carriers [13]. Therefore, GeTe has a high intrinsic carrier concentration of ~10^21^ cm^−3^ at room temperature, resulting in low Seebeck coefficients and high electrical and thermal conductivity [14,15,16]. At present, most studies are focused on element or compound doping in order to optimize the carrier concentration to the optimal range of 10^19^~10^20^ cm^−3^. Doping will introduce a lot of defects which will improve the power factor and reduce thermal conductivity, resulting in an improvement in the thermoelectric performance of GeTe. For example, Bu [17] obtained a *zT* value of 1.1 at room temperature by doping Pb into the GeTe matrix. Researchers have also studied the doping of many other elements, except for Pb because of its toxicity, such as Bi [18,19] or Sb [20,21], which can greatly reduce the carrier concentration of GeTe. Therefore, the thermoelectric performance of the composite is improved by increasing the Seebeck coefficient and decreasing the thermal conductivity. In addition, Mg [22], Cu [23], Sn [24], and Ag [25] doping can also reduce the carrier concentration and thermal conductivity of GeTe. What is more, the thermoelectric properties of GeTe can also be optimized by adjusting the ratio of Ge to Te; the reason for this is that this method can regulate the Ge vacancy concentration [26]. In the area of energy band engineering, the electronic structure can be adjusted by doping elements such as Ca [22], Cd [27], and Mn [28]. In the field of resonant energy levels, the Seebeck coefficient is increased by doping the In [29] element, which distorts the density of states near the Fermi level.

Although great progress has been made in research on GeTe-based TEs, the thermoelectric conversion efficiency is still lower than traditional heat engines. Therefore, it is necessary to further develop GeTe-based TEs with high thermoelectric properties. In this paper, we try to optimize the thermoelectric properties of GeTe by the doping method with Sn, In, and Se and then discuss the effect of multi-doping on the microstructure and thermoelectric properties of GeTe.

## 2. Materials and Methods

### 2.1. Materials and Sample Preparation

First, Ge powder (200 mesh), In powder (200 mesh), Sn powder (200 mesh), Te powder (200 mesh), and Se powder (200 mesh) were weighed according to the stoichiometric ratio of Ge_0.99-*x*_In_0.01_Sn*_x_*Te_0.94_Se_0.06_ (*x* = 0, 0.01, 0.03, 0.06). The weighed powders were mixed in an agate mortar in an argon atmosphere for 2 h. Then, the mixed compounds were compacted into cylindrical pellets using a stainless-steel mold with a diameter of Φ16 mm under a pressure of 8–10 MPa. The pellets were then placed in a quartz tube with a diameter of Φ20 mm and vacuum-sealed under the pressure of ~10^−5^ Pa. Then, the sealed quartz tube was placed in a box-type muffle furnace for melting. The heating rate during synthesis was set to 2 °C/min, and the temperature was raised to 1000 °C for 10 h. After the dwell time, the furnace was allowed to cool naturally. The obtained ingot was ground to powders less than 200 um in order to sinter bulk samples by spark plasma sintering (SPS) under the sintering process conditions of 50 MPa, 525°C, and 10 min. The samples were cut into ~12 × 3 × 3 mm strips for electrical transport property measurements and ~10 × 10 × 2 mm for thermal conductivity and Hall measurements.

### 2.2. Performance Characterization

The crystal structure of the samples was tested by X-ray diffraction (XRD, D/MAX-2550P, Cu-Kα, λ = 0.154056 nm, Rigaku^®^, Tokyo, Japan). The character of the morphology after polishing was tested by SEM (Qutanta FEG 450) with an energy dispersive spectrometer (EDS) to analyze the element distribution.

The electrical properties including the Seebeck coefficient and electrical conductivity were measured using a custom-made testing apparatus developed by the 18th Research Institute of China Electronics Technology Group Corporation. Differential scanning calorimetry (DSC) and thermogravimetric analysis (TG) were carried out using a NETZSCH STA 449 F5 Jupiter instrument. The Hall effect testing apparatus, HET-HT model, was utilized for measuring the carrier concentration of the samples. The thermal diffusivity(*λ*) and specific heat capacity (*C*_p_) of the samples were determined by the laser flash method (NETZSCH^®^, LFA427, Netzschkau, Germany), the density(*ρ*) was measured by the Archimedes’ method, and finally, the thermal conductivity(*κ*) was calculated using the corresponding formula (*κ* = *λ*·*C*_p_·*ρ*).

## 3. Results and Discussion

### 3.1. XRD Result Analysis

**Figure 1:**
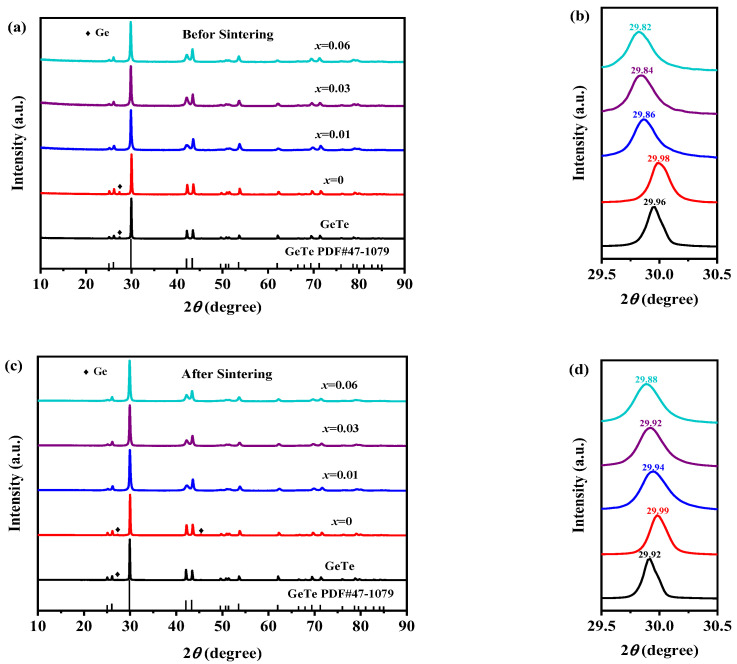
XRD patterns of pure GeTe and Ge_0.99-*x*_In_0.01_Sn*_x_*Te_0.94_Se_0.06_(*x* = 0, 0.01, 0.03, 0.06): (**a**) before sintering and (**c**) after sintering, and the magnification patterns of 29.5~30.5°: (**b**) before sintering and (**d**) after sintering.

The XRD patterns of Ge_0.99-*x*_In_0.01_Sn*_x_*Te_0.94_Se_0.06_(*x* = 0, 0.01, 0.03, and 0.06) and GeTe (a) before sintering and (c) after sintering are presented in Figure 1a. It can be observed that the diffraction peaks of all samples can be indexed by JCPDS#47-1079(*R*3*m*, 160), indicating that all samples belong to the rhombohedral phase structure. In the pure GeTe and the sample (*x* = 0), this is a peak (~27°) corresponding to the Ge element, but it disappears in the samples doped with Sn, indicating that Sn doping facilitates the dissolution of the Ge element. No other impurity peaks were detected.

After magnifying the diffraction peaks in the range of 29.5° to 30.5° for the samples (a) before sintering and (c) after sintering, it can be observed that regardless of the sintering progress, the diffraction peaks shifted to the right at first with the doping of In and Se, and then shifted to the left with the increasing amount of Sn. This can be attributed to the combined doping of In and Se, where the ionic radius of In is larger than Ge, while the ionic radius of Se is smaller than that of Te. At first, the influence of Se becomes more significant because the doping amount of Se is six times that of In, resulting in a rightward shift of the diffraction peak and a decrease in the lattice constant. Moreover, the diffraction peaks shift to the left after the doping of Sn due to the larger ionic radius of it compared to Ge, leading to an increase in the lattice constant. This observation confirms the successful incorporation of In, Se, and Sn elements into the GeTe lattice.

**Figure 2:**
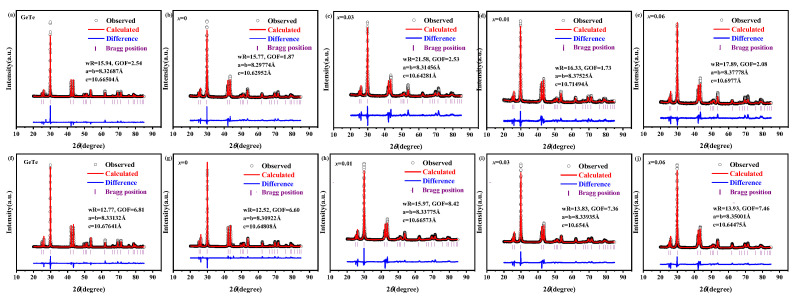
The Rietveld refinements of powder XRD patterns of pure GeTe and Ge_0.99-*x*_In_0.01_Sn*_x_*Te_0.94_Se_0.06_ (*x* = 0, 0.01, 0.03, 0.06): (**a**–**e**) before sintering and (**f**–**j**) after sintering.

Furthermore, the XRD patterns were refined to obtain the lattice constants using GSAS-II software with EXPGUI [30] as presented in Table 1 and Figure 2.

### 3.2. SEM Result Analysis

**Figure 3:**
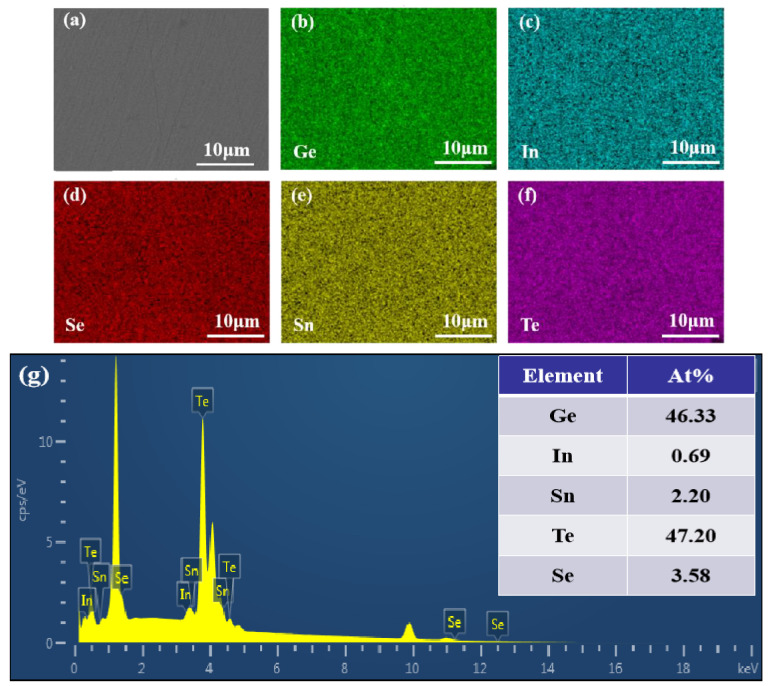
The (**a**) secondary electron SEM photographs, (**b**–**f**) energy dispersive X-ray spectroscopy element mapping of the polished surface, and (**g**) EDS spectrum and atomic percent of Ge_.96_In_0.01_Sn_0.03_Te_0.94_Se_0.06_.

In order to assess the distribution of the Sn element in the sample, the sample with a doping level of *x* = 0.03 was selected for grinding and polishing, followed by SEM and surface-scanning EDS tests. The secondary electron SEM image of the polished surface is shown in Figure 3a, revealing the good quality of the sample surface. Figure 3b–f depicts the surface-scanning elemental distribution maps of the sample (*x* = 0.03), indicating a uniform distribution of all elements within the matrix. The EDS spectrum and atomic percent of Ge_0.96_In_0.01_Sn_0.03_Te_0.94_Se_0.06_ are also shown in Figure 3g, indicating that the target compound was obtained.

### 3.3. Analysis of Electrical Transport Performance

**Figure 4:**
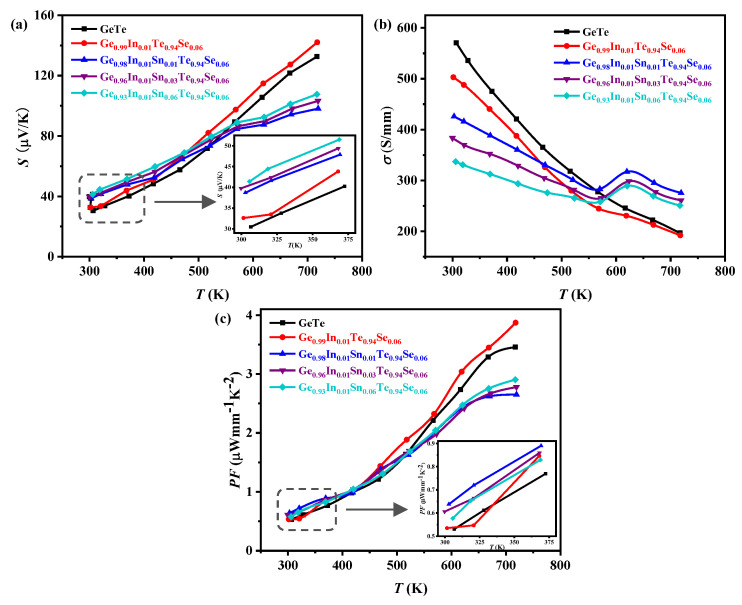
The variation in the (**a**) Seebeck coefficient, (**b**) conductivity, and (**c**) power factor with temperature for the pure GaTe and Ge_0.99-*x*_In_0.01_Sn*_x_*Te_0.94_Se_0.06_ (*x* = 0, 0.01, 0.03, 0.06) samples. The inset shows the magnification patterns of (a) the Seebeck coefficient and (c) the power factor from 300 K to 375 K.

As shown in Figure 4a, all the samples exhibit a positive Seebeck coefficient and increase after doping at room temperature, suggesting a p-type electrical transport behavior. The Seebeck coefficient variation trend of the sample (*x* = 0) is similar to pure GeTe, with an overall improvement in the *S* compared to pure GeTe across the entire temperature range. However, the temperature dependence of the *S* of other samples (*x* = 0.01, *x* = 0.03, *x* = 0.06) shows a significant reduction in the slope of the curve, leading to a lower value for the Sn-doped samples compared to pure GeTe after 580 K.

The variation in electrical conductivity *σ* with temperature for all samples is depicted in Figure 4b. Both pure GeTe and the sample (*x* = 0) exhibit a decreasing tendency in *σ* with the increasing temperature, while the value of the sample (*x* = 0) is lower than GeTe, demonstrating the degenerate semiconductor behavior. In contrast, the *σ* of the Sn-doped samples decreases with the increasing doping level, which is opposite to the trend observed for the *S*. What is more, the *σ* of the Sn-doped samples decreases with the increasing temperature in the range from room temperature to 580 K and then increases slightly before decreasing again with further temperature increase. As a result, the *σ* of the Sn-doped samples eventually becomes higher than that of pure GeTe after approximately 580 K.

The power factor varies with temperature for all samples is presented in Figure 4c, and it exhibits a similar trend to that of the *S*. Compared to pure GeTe, the sample (*x* = 0) shows significant enhancement in the *S* over the entire temperature range, resulting in the maximum power factor achieved at 723 K. However, the samples (*x* = 0.01, 0.03, 0.06) exhibit higher *S* than the other two samples near room temperature but significantly lower values at high temperatures compared to pure GeTe. Consequently, their power factors are lower than that of pure GeTe. This result indicates that In and Se co-doping can improve the electrical transport properties of GeTe, while the co-doping of In, Sn, and Se is detrimental to the high-temperature electrical transport performance of GeTe.

**Figure 5:**
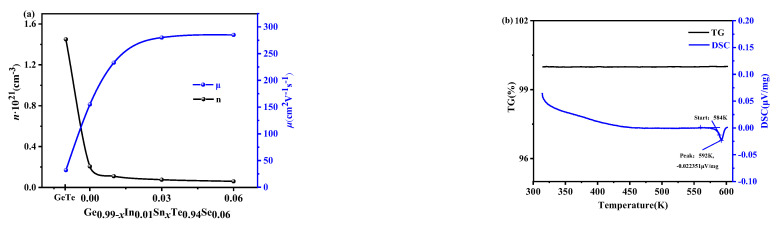
(**a**) The carrier concentration and mobility of GeTe and Ge_0.99-*x*_In_0.01_Sn*_x_*Te_0.94_Se_0.06_(*x* = 0, 0.01, 0.03, 0.06) and (**b**) variation in the DSC and TG with temperature of Ge_0.96_In_0.01_Sn_0.03_Te_0.94_Se_0.06._

Figure 5a shows the variation in carrier concentration and mobility for all samples. The sample (*x* = 0) exhibits a lower carrier concentration than intrinsic GeTe, and as *x* increases, the carrier concentration further decreases while the mobility increases. This observation provides a good explanation for the patterns observed in the *S* and *σ* at room temperature, where the Seebeck coefficient increases with the decreasing carrier concentration while the electrical conductivity decreases. Based on the XRD analysis, it can be concluded that Sn doping promotes the dissolution of Ge and results in reducing the number of vacancies and leads to a significant decrease in carrier concentration. The increase in carrier mobility can be explained by the increase in Sn content, which causes a decrease in Ge vacancy; this change can reduce the probability of hole capture and improve carrier mobility. This ultimately explains the observed changes in the Seebeck coefficient and the electrical conductivity.

In order to analyze the reasons behind the performance change in the Sn-doped samples around 580 K, a differential scanning calorimetry (DSC) analysis was conducted on the samples (*x* = 0.03), and the result are shown in Figure 5b. The DSC curve exhibits a downward peak starting from 584 K, indicating heat absorption. However, the corresponding thermogravimetry (TG) curve remains unchanged. This suggests that the performance changes of the Sn-doped sample around 580 K can be ascribed to a phase transition rather than the volatilization of elements.

**Figure 6:**
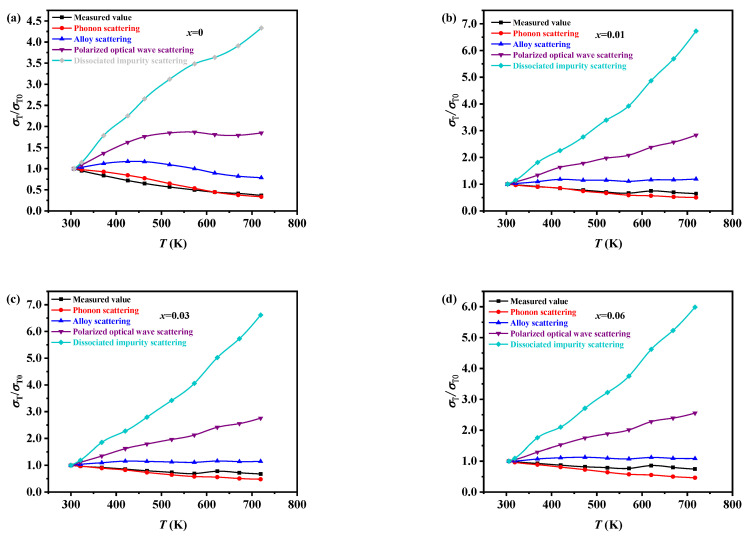
The measured value and the predicted value of the conductivity ratio of Ge_0.99-*x*_In_0.01_Sn*_x_*Te_0.94_Se_0.06_ (**a**) *x* = 0, (**b**) *x* = 0.01, (**c**) *x* = 0.03, and (**d**) *x* = 0.06 vary with temperature under different scattering mechanisms.

The carrier scattering mechanism of the Ge_0.99-*x*_In_0.01_Sn*_x_*Te_0.94_Se_0.06_ (*x* = 0, 0.01, 0.03, and 0.06) samples was calculated using the conductivity ratio method [31] and is depicted in Figure 6. The results indicate that phonon scattering is the dominant mechanism in all samples. Furthermore, alloy scattering becomes more prominent after 550 K, and its strength increases gradually with the increase in *x*.

### 3.4. Analysis of Thermal Transport Performance

**Figure 7:**
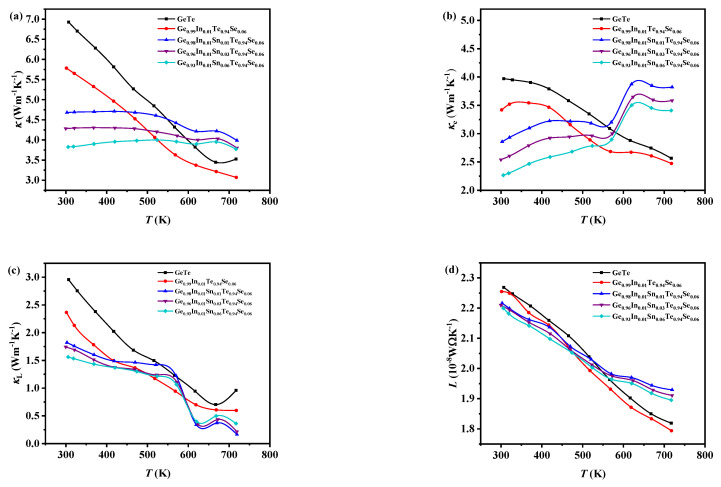
The relationship between (**a**) the total thermal conductivity, (**b**) the carrier thermal conductivity, (**c**) the lattice thermal conductivity, and (**d**) the Lorentz constant with temperature for the pure GeTe and Ge_0.99-*x*_In_0.01_Sn*_x_*Te_0.94_Se_0.06_ (*x* = 0, 0.01, 0.03, 0.06) samples.

The variation in total thermal conductivity (*κ*) with temperature for pure GeTe and the Ge_0.99-*x*_In_0.01_Sn*_x_*Te_0.94_Se_0.06_ (*x* = 0, 0.01, 0.03, and 0.06) samples is depicted in Figure 7a. It should be noted that the *κ* significantly decreases after doping due to the influence of alloy scattering at room temperature. For instance, the *κ* decreases from 6.9 Wm^−1^ K^−1^(GeTe) to 4.6 Wm^−1^ K^−1^ (*x* = 0.06). The *κ* of pure GeTe and the sample of *x* = 0 decreases with the increasing temperature up to 700 K, while the variation in the doped samples is minimal and exhibits a small fluctuation around 580 K, which is related to the phase transition. As a result, the *κ* of the Sn-doped samples is higher than that of the pure GeTe at high temperatures.

The carrier thermal conductivity (*κ*_e_) of the samples shown in Figure 7b can be calculated by the formula:

*κ*_*e*_ = *LσT* (4)

*L* = 1.5 + exp(−*S*/116) (5)

where *L* is the Lorenz constant and the results are presented in Figure 7d. The *κ*_e_ of GeTe and the sample of *x* = 0 are reduced with the increasing temperature. The Sn-doped samples show a slight reduction in carrier thermal conductivity with increasing *x*, and there is an increasing trend with temperature, particularly after 580 K. This observation also indicates the occurrence of a phase transition near 580 K, leading to changes in the crystal structure and carrier concentration. The results indicate that the variation in *κ*_e_ with temperature is similar to the electrical conductivity, suggesting a strong correlation between the two parameters.

The lattice thermal conductivity (*κ*_L_) shown in Figure 7c can be obtained by Formula (3). It can be observed that the variation in *κ*_L_ with temperature for the pure GeTe and *x* = 0 samples is generally consistent with the variation in *κ* with temperature. The *κ*_L_ of the *x* = 0.01, 0.03, and 0.06 samples exhibits minimal changes with temperature increase before 580 K, followed by a sharp decrease. This further confirms the occurrence of a phase transition around 580 K, which increases the defect concentration and enhances phonon scattering, leading to a drastic reduction in lattice thermal conductivity.

**Figure 8:**
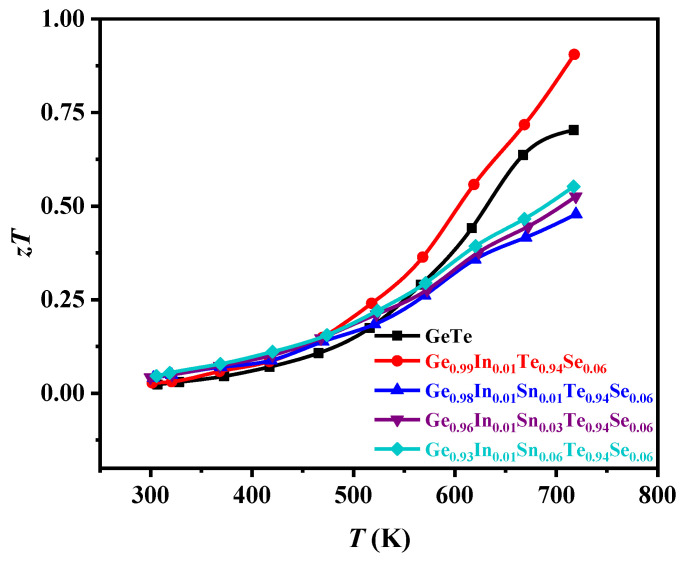
The relationship between the *zT* values of the pure GeTe and Ge_0.99-*x*_In_0.01_Sn*_x_*Te_0.94_Se_0.06_ (*x* = 0, 0.01, 0.03, and 0.06) samples with temperature.

Finally, the *zT* values of the pure GeTe and Ge_0.99-*x*_In_0.01_Sn*_x_*Te_0.94_Se_0.06_(*x* = 0, 0.01, 0.03, and 0.06) samples prepared by SPS as a function of temperature were obtained and are shown in Figure 8. The inset shows the magnification patterns of the *zT* value from 300 K to 375 K.

The *zT* value of the sample (*x* = 0) was optimized in the whole temperature range, and the maximum *zT* value is 0.91 at ~723 K. However, the *zT* values of the samples of Ge_0.99-*x*_In_0.01_Sn*_x_*Te_0.94_Se_0.06_ (*x* = 0.01, 0.03, and 0.06) are lower than those of the pure GeTe because of the lower power factor and the higher thermal conductivity of the samples at high temperatures. It can be seen that In, Sn, and Se co-doping has no effect on improving the thermoelectric properties of GeTe at higher temperatures. The most important aspect is that the synergistic effect of multi-element doping is not the sum of all the effects of single-element doping but rather a comprehensive reflection of the interaction between elements.

## 4. Conclusions

In this paper, GeTe and Ge_0.99-*x*_In_0.01_Sn*_x_*Te_0.94_Se_0.06_ (*x* = 0, 0.01, 0.03, and 0.06) samples were prepared by vacuum synthesis plus spark plasma sintering. The samples were characterized by XRD, SEM, DSC/TG, scattering mechanism analysis, and thermoelectric property tests. The XRD results showed that these samples are all diamond-shaped phases. The In, Sn, and Se elements were all doped into the lattice of GeTe, and the solid solution limit was not reached. The peaks of Ge disappear after doping with Sn, which indicates that Sn doping is beneficial to the dissolution of the Ge element. The SEM photographs and EDS results show that the elements are distributed in the matrix uniformly. The main scattering mechanism is phonon scattering, which is enhanced with the increase in the Sn doping amount. The thermoelectric properties of GeTe are optimized due to the introduction of resonant energy levels by In doping and increased point defects by Se doping to enhance phonon scattering. The change tendency with the temperature of the Seebeck coefficient, electrical conductivity, and thermal conductivity of the Sn-doped samples obviously slowed down, and the temperature at which the phase transition occurs decreased. Therefore, the *zT* value of the Sn-doped samples is higher at room temperature to ~580 K and lower at higher temperatures compared with GeTe. Finally, the *zT*_max_ was obtained in Ge_0.99_In_0.01_Te_0.94_Se_0.06_ at the temperature of ~723K.

## Figures and Tables

**Table 1:** Lattice constants and cell volumes of pure GeTe and Ge_0.99-*x*_In_0.01_Sn*_x_*Te_0.94_Se_0.06_(*x* = 0, 0.01, 0.03, 0.06) before and after SPS sintering.

	Sample	Lattice Constants (a/Å)	Lattice Constants (c/Å)
After Synthesis	GeTe	8.32687	10.66504
	Ge_0.99_In_0.01_Te_0.94_Se_0.06_	8.29774	10.62952
	Ge_0.98_In_0.01_Sn_0.01_Te_0.94_Se_0.06_	8.31456	10.64281
	Ge_0.96_In_0.01_Sn_0.03_Te_0.94_Se_0.06_	8.37525	10.71494
	Ge_0.93_In_0.01_Sn_0.06_Te_0.94_Se_0.06_	8.37778	10.69770
After Sintering	GeTe	8.33132	10.67641
	Ge_0.99_In_0.01_Te_0.94_Se_0.06_	8.30922	10.64808
	Ge_0.98_In_0.01_Sn_0.01_Te_0.94_Se_0.06_	8.33775	10.66573
	Ge_0.96_In_0.01_Sn_0.03_Te_0.94_Se_0.06_	8.33935	10.65400
	Ge_0.93_In_0.01_Sn_0.06_Te_0.94_Se_0.06_	8.35001	10.64475

## Data Availability

The data presented in this study are available from the corresponding author upon request.
